# A call for the United States to continue investing in science

**DOI:** 10.1128/mbio.00648-25

**Published:** 2025-02-27

**Authors:** Ira Blader, Felicia Goodrum, Michael J. Imperiale, Arturo Casadevall, Cesar A. Arias, Andreas Baumler, Carey-Ann D. Burnham, Christina A. Cuomo, Corrella S. Detweiler, Graeme N. Forrest, Jack A. Gilbert, Susan Lovett, Stanley Maloy, Alexander McAdam, Irene Newton, Gemma Reguera, George A. O'Toole, Patrick D. Schloss, Ashley Shade, Marvin Whiteley

**Affiliations:** 1 Biomedical Sciences and Pathobiology Virginia-Maryland College of Veterinary Medicine 70732 , Blacksburg, Virginia, USA; 2 Department of Immunology, The University of Arizona 8041 , Tucson, Arizona, USA; 3 Department of Microbiology and Immunology, University of Michigan 1259 , Ann Arbor, Michigan, USA; 4 Johns Hopkins Bloomberg School of Public Health 25802 , Baltimore, Maryland, USA; 5 Center for Infectious Diseases, Houston Methodist Hospital, Houston, Texas, USA; 6 Department of Medicine, Weill Cornell Medical College, New York, New York, USA; 7 Medical Microbiology and Immunology, University of California, Davis 8789 , Davis, California, USA; 8 Pattern Bioscience, Austin, Texas, USA; 9 Molecular Microbiology and Immunology, Brown University 6752 , Providence, Rhode Island, USA; 10 MCD-Biology University of Colorado Boulder 1877 , Boulder, Colorado, USA; 11 Infectious Diseases Rush University Medical Center 2468 , Chicago, Illinois, USA; 12 University of California San Diego 8784 , La Jolla, California, USA; 13 Brandeis University 8244 , Waltham, Massachusetts, USA; 14 Biology Department, San Diego State University 7117 , San Diego, California, USA; 15 Department of Laboratory Medicine, Boston Children's Hospital 1862 , Boston, Massachusetts, USA; 16 Indiana University, Bloomington 1771 , Bloomington, Indiana, USA; 17 Michigan State University, East Lansing, Michigan, USA; 18 Geisel School of Medicine at Dartmouth, Hanover, New Hampshire, USA; 19 Microbiology & Immunology University of Michigan 1259 , Ann Arbor, Michigan, USA; 20 CNRS Delegation Alpes, Lyon, Rhône-Alpes, France; 21 Georgia Institute of Technology 1372 , Atlanta, Georgia, USA

**Keywords:** research investment, scientific advances, innovation

## EDITORIAL

The U.S. life science research mission is critical not only to human health and understanding the natural world but also to agriculture and food production, technological innovations, socioeconomic progress, and our national defense and leadership worldwide. A 2025 Research!America survey reveals that 92% of Americans want government to actively work to promote medical progress, in part by funding infectious and chronic disease research. Why? Because biomedical research saves lives, prevents suffering, and increases quality of life for not only Americans but for people throughout the world. While less well appreciated, science also drives enormous economic growth. Indeed, historically there has been widespread bipartisan support for biomedical funding by the federal government. Below we discuss the U.S. scientific research enterprise and provide evidence and arguments we hope the ASM community can use to advocate for science.

Since 1945, science and technology have driven 85% of the economic growth in America. U.S. prosperity following World War II was in large part due to the investment in science and technology, spurred on largely by Vannevar Bush’s recommendation that the country invest in fundamental research at academic institutions. This investment is the foundation of not only our citizens’ well-being but also our security. Currently, science contributes strongly to economic prosperity, as each dollar funded by the National Institutes of Health (NIH) and other government entities leads to an average of $2.46 in economic activity, creating jobs and supporting infrastructure. U.S. support for biomedical research has contributed to some of the most important advances to treat patients (see Table 1 for a partial list of such advances in the past 30 years). And this is not limited to biomedical discovery; advances in other areas, including agriculture, materials, computing/artificial intelligence, transportation, and communications, have been due to government investments in research performed at universities and other non-profit institutions. Furthermore, 9 in 10 Americans want the United States to be a global leader in science and technology. A majority across the political spectrum support more federal investments in science and technology to ensure that the United States remains a global leader in innovation. However, many in the survey (78% of Americans) are concerned about the standing of the United States as a world leader in science.

**TABLE 1:** Highlights of U.S. government-funded advances in biomedical sciences/healthcare

Year	Development^*a*^	Outcome
1990–2003	Human Genome Project	Personalized medicine (e.g. targeted therapies for breast cancer, including HER2 inhibitors like Herceptin, and chronic myeloid leukemia [Gleevec])
1990–present	Targeted cancer therapies	Immunotherapy (e.g. checkpoint inhibitors like Keytruda and Opdivo)
1995	Effective therapy for HIV	Reduced mortality; reduced transmission
1998	mAb to RSV	Prevention of disease in infants at risk
1998	First drug therapy for hepatitis B virus	Reduced risk of disease progression
2000	Pneumococcal conjugate vaccine	Reduction in childhood otitis media and invasive disease
2000	Stem cell therapy	Pluripotent stem cells for treatment of spinal cord injuries, macular degeneration, etc.
2005	Meningococcal conjugate vaccine	Reduction in meningitis cases
2006	Human papillomavirus vaccine	Reduction in cervical cancer
2006	Rotavirus vaccine	Reduction in childhood diarrhea
2012	CRISPR	Gene editing of heritable genetic diseases
2012	First home test for HIV infection	Early diagnosis reduces spread and leads to early therapy
2014	Antivirals for hepatitis C virus	HCV becomes a curable disease
2014	BRAIN initiative	Brain imaging and neural interface technology
2017	Shingrix vaccine	Shingles prevention
2017	Artificial pancreas	Automated insulin delivery system for type 1 diabetes
2017	CAR-T	Cell therapy for B-cell leukemia, lymphoma, multiple myeloma
2019	mAb therapies for Ebola virus	Reduced mortality
2020–2021	mAbs, vaccines, antivirals, rapid tests for SARS-CoV-2	Reduced mortality
2021	Long-acting therapy for HIV	Ease of use, reduced transmission, reduced mortality
2021	First effective malaria vaccine	Reduced malaria cases
2023	RSV vaccine	Reduced mortality and morbidity

^*a*^ mAb, monoclonal antibody.

Funding cuts proposed in 2025, and in part executed through U.S. presidential executive orders (EOs), will have undecidedly negative effects that will constrain the American scientific enterprise. The rapidity of the release of these EOs has impacted the mission and focus of federal and academic scientific workforces who are dedicated to improving health and welfare of the United States. Our major federal life science funding agencies (including NIH, National Science Foundation [NSF], U.S. Departments of Energy and Agriculture, and others) have already felt the impacts, most immediately chaos in the grant submission/review process and ongoing funding mechanisms and more recently with dramatic reductions in these agencies’ workforces. This represents a striking reversal of policies that are the bedrock of the U.S. scientific research enterprise and its leadership in the world. These have been met with a number of legal challenges. Some have received temporary restraining order protections, and others are pending judicial decisions.

Basic science has historically received strong bipartisan support, a testament to its importance to the fabric of America. Spending on science in the United States accounts for just over 1% of all federal spending such that deep cuts will not impact the budget deficit but will have a striking impact on a valuable enterprise that makes America strong and secure. Further, the position of the United States as the world’s leading investor in scientific research and development was already slipping due to large investments by the Chinese government. U.S. spending as a percentage of gross domestic product has fallen from 1.9% to 0.9% in the last 50 years, and China now leads the world in the most-cited publications and the number of patents in this field. Now is not the time to further reduce or constrain the scientific enterprise, as federal funding for U.S. science agencies already sits at a 25-year low.

One justification that has been posited for cutting back public funding for research is the belief that biomedical research should be driven by the private sector. While its contribution to scientific discoveries is undeniable, the private sector is neither designed nor equipped for basic biomedical research nor to support research into rare diseases whose treatments would not be profitable. The U.S. government funded many of the initial discoveries that led to the development of countless therapies to treat some of our most important diseases. Government-supported studies focused on how T cells become exhausted, which led to the development of checkpoint inhibitors to treat cancers. Similarly, personalized medicine is the direct result of the support by the U.S. government and other entities not only in sequencing the human genome but also in the decades-long work in developing the tools that enabled the project to be performed. Moreover, basic research with no obvious role in human health at the time it was being performed has delivered major human health rewards. This includes the genome editing tool, CRISPR, that was originally discovered as a mechanism that bacteria use to ward off viral infections. CRISPR is already being used to treat sickle cell anemia and will likely be used in the near future to treat diseases including amyotrophic lateral sclerosis (ALS), hemophilia, Duchenne muscular dystrophy, cancer, and diabetes. Finally, NIH’s investment in research on rare diseases such as myasthenia gravis, leukodystrophy, Ehlers-Danlos syndrome, and Rett syndrome, among others, cannot be duplicated by the private sector because researching, let alone treating, these disorders is not a profitable venture.

Current funding models have two components–direct and indirect costs. Direct costs pay for the equipment, supplies, reagents, and salaries. The last includes not only the principal investigators who write grant applications and direct the projects but also the trainees (e.g., students and postdoctoral fellows) and staff that perform the majority of the experiments. Indirect costs pay for substantial infrastructure costs that support the funded research. Cuts in indirect costs will result in the loss of many ancillary jobs, including accountants, administrative assistants, building maintenance workers, custodians, regulatory monitors, and IT professionals. These job losses will have a negative effect on the U.S. economy. For example, Texas receives $1.85 billion in NIH awards that directly supports over 29,500 jobs in research. However, these funds also generate a bioscience industry impact in Texas of an additional 112,000 jobs and 7,300 businesses. The economic activity returned on this investment is $5.8 billion for the state, over $3 for every $1 invested by the U.S. government. In Alabama, The University of Alabama at Birmingham is the state’s largest employer, and relies on over $400 million from the NIH. Finally, losses of both direct and indirect costs will not only impact basic and clinical research but also significantly impede academic medical centers and institutes from conducting clinical drug trials, and this will result in significant delays in advancing new drugs and therapies from being approved and employed.

Besides impacting current research efforts, these proposed changes to publicly supported research will deter the next generation of scientists from entering the field. And at a time when the United States ranks 14th in researchers relative to the overall labor force, further reductions in training will have significant consequences to our future scientific progress. Scientific training is an apprenticeship system where trainees work alongside and learn from their mentors who are the principal investigators of federally funded research and are seasoned in the art of experimentation. Training a scientist can take a decade or more. These trainees be will not only our future leaders, who run their own laboratories, but also our health care providers, private sector researchers, scientific policy experts, first responders to infectious disease outbreaks, public health officers, and countless other professions. Advanced training for these professions is not only textbook-based learning but must also include training in how to design and execute experiments as well as critically analyze and interpret data. Critical evaluation of laboratory science is a skill that physicians also employ in properly diagnosing and selecting appropriate treatment regimens. Discouraging or constraining the next generation of scientists from entering the field to serve our country will have long term negative impacts on U.S. leadership in science worldwide and will diminish the advancement of human health that depends on the scientific enterprise.

Further contractions to research funding and the scientific workforce will lead to increases in infant mortality, illnesses, and death due to otherwise preventable diseases, prevent the United States from facing and responding to new and emerging health challenges, and stifle the discoveries and innovations that fuel future biomedical advances. As ASM journal editors, we call on all members of ASM to push back against attacks on the U.S. scientific enterprise. Our power stands in a concerted, united effort to educate our citizens about the negative effects these actions will have on their everyday lives and the lives of their loved ones. We hope you will carry these arguments into your communities. We applaud efforts of our colleagues who have already contacted their elected officials, spoken out publicly against these actions, and engaged the public in how U.S. government support of publicly funded research is vital to the success of the United States. As journal editors, our role in this response is to provide platforms for scientists to communicate with the public, help our scientific societies partner with their membership, and continue to publish rigorous and impactful research that will advance the microbial sciences and grow trust within the American public. We will not back down from our roles in this fight and we implore our colleagues help us all keep American research great and prosperous.

## AFTER EPUB

*[This article was published on 27 February 2025 with errors in the byline. The byline was corrected in the current version, posted on 11 March 2025.]*

## EDITOR'S NOTE

**Ed. Note:** This editorial is being published by the following ASM journals: *Antimicrobial Agents and Chemotherapy, Applied and Environmental Microbiology, ASM Case Reports, Clinical Microbiology Reviews, Infection and Immunity, Journal of Bacteriology, Journal of Clinical Microbiology, Journal of Microbiology and Biology Education, Journal of Virology, Microbiology and Molecular Biology Reviews, Microbiology Resource Announcements, Microbiology Spectrum, mBio, mSphere,* and *mSystems.*

